# High T2-weighted signal intensity is associated with myocardial deformation in hypertrophic cardiomyopathy

**DOI:** 10.1038/s41598-019-39456-z

**Published:** 2019-02-25

**Authors:** Ruo-yang Shi, Dong-aolei An, Bing-hua Chen, Rui Wu, Chong-wen Wu, Liang Du, Jiong Zhu, Meng Jiang, Jian-rong Xu, Lian-ming Wu

**Affiliations:** 1 Department of Radiology, Ren Ji Hospital, School of Medicine, Shanghai Jiao Tong University, Shanghai, China; 2 Robotics Institute, Shanghai Jiao Tong University, Shanghai, China; 3 Department of Cardiology, Ren Ji Hospital, School of Medicine, Shanghai Jiao Tong University, Shanghai, China

## Abstract

The association between global and segmental myocardial strain impairment and fibrosis extent in hypertrophic cardiomyopathy (HCM) is widely verified. The aim of this study was to investigate the contribution of high T2-weighted signal intensity (HighT2) to myocardial deformation in HCM. We prospectively recruited 57 patients with HCM examined by a 3.0 Tesla magnetic resonance scanner with cine, T2-weighted imaging with fat saturation and phase-sensitive inversion recovery. Global and segmental radial, circumferential and longitudinal strains were included for analysis. The extent of HighT2 was negatively correlated with global radial strain (ρ = −0.275, p = 0.038) and positively correlated with global circumferential strain (ρ = 0.308, p = 0.02) and global longitudinal strain (ρ = 0.422, p = 0.001). Radial, circumferential and longitudinal strains were all significantly associated with segment thickness. Regarding circumferential strain, segments at the mid-ventricular level with LGE and HighT2 showed more impairment than segments with only LGE. For longitudinal strain, the influence of HighT2 appeared only at the mid-ventricular level. The HighT2 extent in HCM was observed to contribute to global and segmental strain parameters. At the segmental level, HighT2 indeed affects left ventricular deformation, and follow-up studies are still warranted.

## Introduction

Hypertrophic cardiomyopathy (HCM) is characterized by heterogeneous left ventricular hypertrophy (LVH) and variability in both clinical presentation and disease course^1^. Although in the majority of HCM patients global systolic function is usually preserved, the contractility and extensibility of the individual cardiac myocytes are most likely compromised. The thickness and morphology of cardiac tissue affected by HCM decreases myocardial strain^2^. Researches also suggest that both heterogeneous hypertrophy and fibrosis contribute to left ventricular (LV) myocardial deformation^3,4^. Moreover, cardiac magnetic resonance (CMR) with contrast identified that late gadolinium enhancement (LGE) increased the risk of future adverse events^5,6^.

Myocardial strain, a precise method for quantifying LV function, has been widely used with speckle tracking echocardiography (STE). Global and segmental strain impairments observed in HCM have been verified by STE^7,8^. Over the past decade, CMR has been used to measure cardiac muscle deformation. The CMR tagging technique was among the first techniques used and soon became validated to assess myocardial strain; in addition, it has good intra-observer agreement^9^. However, the CMR tagging technique is time consuming and has low-grade spatial resolution. Tissue tracking with conventional cine imaging is well suited to quantify the myocardial global and segmental strain^10,11^. Longitudinal strain is computed by using long-axis images, whereas radial and circumferential strains are estimated using short-axis images. Tissue shortening in the circumferential and longitudinal directions is negative, while ventricular wall thickening in the radial plane is positive. Notably, several studies have found a positive correlation between strain parameters measured with STE and CMR tissue tracking^12,13^.

CMR with LGE can identify myocardial fibrosis in HCM. The extent of LGE was associated with sudden death^14^ and tachyarrhythmia^15^, but LGE cannot distinguish between acute and chronic disorders^16^. The relation between the extent of focal fibrosis and myocardial deformation has been proved by previous research, and the association between the extent of diffuse fibrosis assessed by T1 mapping and strain has been reported^17^. However, high T2-weighted signal intensity (HighT2) has been shown to increase the risk of sudden death in HCM patients^18^ and is associated with active tissue injury^19^. The correlation of HighT2 areas with myocardial deformation has not been studied before. Thus, the purpose of this study was to investigate the contributions of HighT2 areas to the global and segmental deformation in HCM patients by using CMR tissue tracking.

## Methods

The study was approved by the institutional review board of Ren Ji Hospital, and written informed consent were obtained from all subjects. All methods were performed in accordance with the relevant guidelines and local regulations.

### Study population

Sixty-four adults with HCM were enrolled from August 2017 to March 2018 at Ren Ji Hospital. The inclusion criteria were as follows: patients with a diagnosis of HCM confirmed by echocardiography or CMR and without systemic disease. Renal function was evaluated by estimated glomerular filtration rate (eGFR) within 3 days prior to the CMR examination. Only patients with eGFR >30 mL/min/1.73 m^2^ could receive CMR examination.

Patients with hypertension were carefully reviewed his/her medical history and CMR images (n = 11). The exclusion criteria were as follows: (1) prior history of significant coronary artery disease (n = 4); (2) previous heart surgery or any interventional therapy (n = 2); or (3) athletic training (n = 1). Ultimately, this prospective study included 57 HCM patients with CMR examinations. Of all the 57 patients, 24 (42%) were taken β-blocker and 14 (24%) were taken Calcium-antagonist for therapy. The clinical characteristics of the patients are summarized in Table 1.

**Table 1:** Clinical and CMR parameters of HCM patients.

	HCM patients (n = 57)
Age	52.93 ± 15.99
Gender (Male/Female)	38/19
Body surface area (m^2^)	1.81 ± 0.22
**Symptom**	
Chest pain	24 (42.1%)
NYHA class (I~II/III~IV)	45/12
**Pattern of hypertrophy**	
Septal asymmetrical	41
Concentric	8
Predominantly midcavity	3
Apical	4
Lateral asymmetrical	1
**CMR parameters**	
EDV (ml)	123.10 ± 27.28
ESV (ml)	56.20 ± 20.28
LVEF (%)	73.80 ± 6.92
LVMi (ml/m^2^)	100.50 ± 32.91
LVOT Obstruction	23
HighT2 presence patients	23 (40.4%)
HighT2 extent (% of LV mass)	13.10 ± 10.38
HighT2 extent (segments)	3.12 ± 2.66
LGE presence patients	41 (71.9%)
LGE extent (% of LV mass)	16.39 ± 13.20
LGE extent (segments)	4.75 ± 3.92
Radial strain (%)	35.42 ± 12.86
Circumferential strain (%)	−16.79 ± 4.66
Longitudinal strain (%)	−12.88 ± 3.52

NYHA: New York Heart Association; EDV: end diastolic volume; ESV: end systolic volume; LVEF: left ventricular ejection fraction; LVMi: Left ventricular mass index; LVOT: left ventricular outflow tract; HighT2: high signal intensity on T2 weighted imaging; LGE: late gadolinium enhancement.

### CMR imaging

CMR examinations were performed with a 3.0 Tesla scanner (Ingenia, Philips Healthcare, Best, The Netherlands) and a dS Torso coil anterior to the chest. All images were acquired with repeated breath holding and ECG gating. The images obtained included short-axis stacks from base to apex of the left ventricle and long-axis (3-, 4-, and 2-chamber views) balanced steady-state free precession (b-SSFP) cine sequences. Short-axis T2-weighted short-time inversion recovery (T2-STIR) and 2D phase-sensitive inversion recovery (PSIR) sequences were matched with the cine images. PSIR images were acquired 10–13 minutes after bolus injection of 0.15 mmol/kg gadolinium-DTPA (Magnevist Bayer Healthcare, Berlin, Germany). The sequences of the technique performed are as follows: (1) b-SSFP cine sequences: repetition time (TR) = 2.8 ms, echo time (TE) = 1.4 ms, slice thickness = 7 mm, slice gap = 3 mm, acquired matrix = 1.2 mm × 1.2 mm, phases per cardiac cycle = 30, field of view (FOV) = 300 mm × 300 mm; (2) T2-STIR sequence: TR = 1714 ms, TE = 75 ms, FOV = 300 mm × 300 mm, acquired matrix = 0.89 mm × 0.89 mm; and (3) PSIR sequence: TR = 6.1 ms, TE = 3 ms, FOV = 300 mm × 300 mm, acquired matrix = 1.6 mm × 1.9 mm.

### Image post-processing

CMR image post-processing was performed with cvi42 (Circle Cardiovascular Imaging Inc., Calgary, Canada). A radiologist with 3 years of experience (RY. S) and a radiologist with 4 years (DAL. A) of experience separately analysed all CMR images and subsequently discussed and settled any uncertainties or discrepancies with a cardiologist with 20 years of experience and 6 years of CMR experience (M. J). Short-axis cine images were imported to the Short 3D module for left ventricular ejection fraction (LVEF) and myocardium mass calculation. Short-axis and 3 long-axis cine images were imported to a Tissue Tracking module for global and segmental strain analysis. The systolic and diastolic parameters of radial, circumferential, and longitudinal strain (RS, CS, and LS, respectively) were calculated, global and 16 segmental strain parameters were computed. T2-STIR and PSIR sequences were analysed by a tissue characterization module. The extents of HighT2 and LGE are summarized by the percentage of LV mass and the number of involved segments, respectively.

### Statistical analysis

Numerical data are presented as the means ± standard deviations. Spearman’s correlation analysis was used to analyse the association of the extents of HighT2 and LGE with strain parameters. One-way analysis of variance with Tukey’s multiple comparisons test or the Kruskal-Wallis analysis with Dunn’s multiple comparisons test was employed for comparisons among segments with and without LGE and/or HighT2. For the gradient of the LV strain from base to apex, a sub-group analysis proceeded separately at the basal, mid-ventricular and apical levels. Linear regression model was used for multivariate analysis of all segments, segments ≥15 mm, and segments <15 mm. All statistical analyses were performed with SPSS (version 22, Chicago, IL) and GraphPad Prism (version 7, GraphPad Software, La Jolla, California, USA).

## Results

### Global analysis

A total of 57 HCM patients (38 male and 19 female patients) were included in this study. The 57 patients had predominately septal HCM (n = 41), followed by concentric and diffuse HCM (n = 8), apical HCM (n = 4), mid-cavity HCM (n = 3) and lateral HCM (n = 1). The basic CMR parameters of LV are summarized in Table 1.

HighT2 presences were observed in 23(40.4%) patients and LGE presences were observed in 41(71.9%) patients. The Spearman correlations of LV mass and the extents of HighT2 and LGE with global strain parameters determined by tissue tracking are displayed in Table 2. The global radial strain (GRS), global circumferential strain (GCS) and global longitudinal strain (GLS) were significantly correlated with LV mass, LGE extent and HighT2 extent. The HighT2 extent was negatively correlated with GRS (ρ = −0.275, p = 0.038) and positively correlated with GCS (ρ = 0.308, p = 0.02) and GLS (ρ = 0.422, p = 0.001).

**Table 2:** Correlation of HighT2/LGE extent with strain parameters.

	LVM		LGE extent		HighT2 extent	
	Correlation coefficient	P value	Correlation coefficient	P value	Correlation coefficient	P value
GRS	−0.572	<0.001	−0.364	0.005	−0.275	0.038
GCS	0.411	0.002	0.360	0.006	0.308	0.02
GLS	0.601	<0.001	0.540	<0.001	0.422	0.001

LVM: Left ventricular mass; LGE: late gadolinium enhancement; HighT2: high signal intensity on T2 weighted imaging; GRS: global radial strain; GCS: global circumferential strain; GLS: global longitudinal strain.

### Segmental analysis

**Figure 1:**
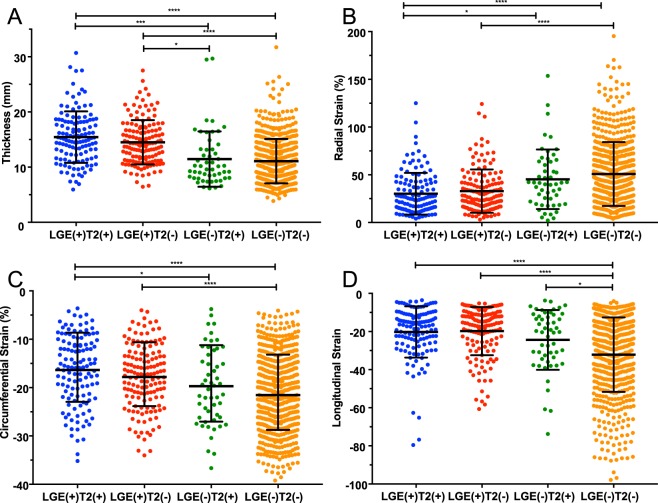
Post hoc comparison of segments with and without LGE and/or HighT2. The asterisks represent the p values as follows: *P ≤ 0.05; **P ≤ 0.01; ***P ≤ 0.001; ****P ≤ 0.0001. (**A**) Comparison of segment thicknesses. (**B**) Comparison of radial strains. (**C**) Comparison of circumferential strains. (**D**) Comparison of longitudinal strains.

The results of the segmental analysis were divided into four groups according to the presence of LGE/HighT2 in 912 segments from the 57 patients. Most HighT2-positive segments were accompanied by LGE (126 of 178 segments). Neither HighT2 nor LGE was observed in 589 segments. The strain parameters and thickness comparison are summarized in Table 3 and Fig. 1. Segments with LGE presence were significantly thicker and showed lower strain in all directions compared with segments without LGE. In the segments with LGE presence, the HighT2 group was not found to be different from the group without HighT2. However, the average wall thickness of only HighT2 presence group was significantly thinner than those of LGE presences groups. The LS of the HighT2-only group was lower to that of the non HighT2 or LGE presence group.

**Table 3:** Comparison of segments with and without LGE and/or HighT2.

In all	LGE (+)		LGE (−)		P value
	High T2 (+) (n = 126)	High T2 (−) (n = 145)	High T2 (+) (n = 52)	High T2 (−) (n = 589)	
Thickness	15.44 ± 4.66	14.50 ± 4.02	11.45 ± 5.01	11.08 ± 4.02	<0.001
RS	30.17 ± 21.81	32.92 ± 22.68	45.29 ± 31.21	50.78 ± 33.47	<0.001
CS	−16.37 ± 7.15	−17.80 ± 6.61	−19.70 ± 7.90	−21.54 ± 7.78	<0.001
LS	−20.23 ± 13.45	−19.80 ± 12.67	−24.43 ± 15.69	−32.15 ± 19.56	<0.001

HighT2: high signal intensity on T2 weighted imaging; LGE: late gadolinium enhancement; RS: radial strain; CS: circumferential strain; LS: longitudinal strain.

**Figure 2:**
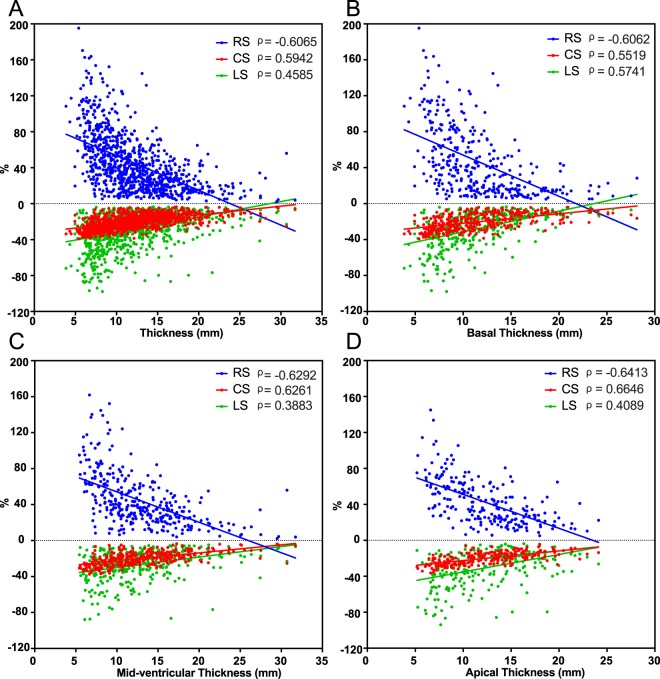
Correlations of segment thicknesses and 3 strain parameters. (**A**) Correlation of all segments and thicknesses. (**B**) Correlation of basal segments and thicknesses. (**C**) Correlation of mid-ventricular segments and thicknesses. (**D**) Correlation of apical segments and thicknesses.

The correlations of segment thickness and strain parameters are indicated in Fig. 2. RS, CS and LS are all significantly associated with segment thickness.

**Figure 3:**
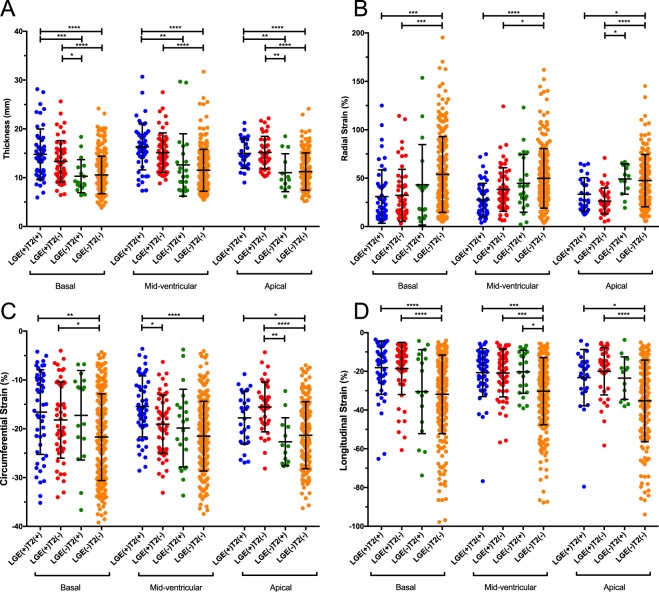
Post hoc comparison of segments with and without LGE and/or HighT2 at the basal, mid-ventricular and apical levels. The asterisks represent the p values as follows: *P ≤ 0.05; **P ≤ 0.01; ***P ≤ 0.001; ****P ≤ 0.0001. (**A**) Comparison of segment thicknesses. (**B**) Comparison of radial strains. (**C**) Comparison of circumferential strains. (**D**) Comparison of longitudinal strains.

Considering the gradient of the LV strain from base to apex, segments were separately analysed at the basal, mid-ventricular and apical levels. The correlations of the strain parameters and thicknesses at the 3 levels are displayed in Fig. 2. At the 3 levels, the RS, CS and LS were all significantly correlated with segment thicknesses. The strain parameters and thickness of groups with and without LGE and/or HighT2 at the 3 levels are shown in Table 4 and Fig. 3. At all levels, the wall thicknesses of the HighT2-only group were thinner than that of the LGE groups, and LGE segments with or without HighT2 showed no difference in thickness. LGE presence impaired all the strain parameters at the 3 levels. For HighT2 results in 3 levels, the presence of HighT2 did not affect the RS, and for CS, segments with LGE and HighT2 were more impaired than segments with only LGE present at the mid-ventricular level. Regarding LS, the influence of HighT2 only appeared at the mid-ventricular level, and HighT2 presence reduced LS compared with negative segments.

**Table 4:** Comparison of segments with and without LGE and/or HighT2 in basal, mid-ventricular and apical level.

Basal	LGE (+)		LGE (−)		P value
	High T2 (+) (n = 49)	High T2 (−) (n = 52)	High T2 (+) (n = 17)	High T2 (−) (n = 224)	
Thickness	14.78 ± 5.20	13.34 ± 4.26	10.30 ± 3.39	10.56 ± 3.85	<0.001
RS	31.03 ± 27.62	32.23 ± 26.95	43.17 ± 41.58	53.87 ± 39.14	<0.001
CS	−16.57 ± 8.68	−18.20 ± 7.81	−17.24 ± 9.18	−21.71 ± 8.89	<0.001
LS	−18.10 ± 13.80	−18.51 ± 13.46	−30.51 ± 21.66	−31.86 ± 20.32	<0.001
**Mid-ventricular**	**High T2 (+) (n = 50)**	**High T2 (−) (n = 54)**	**High T2 (+) (n = 22)**	**High T2 (−) (n = 216)**	**P value**
Thickness	16.35 ± 4.76	15.13 ± 4.06	12.60 ± 6.40	11.52 ± 4.29	<0.001
RS	27.60 ± 17.16	38.30 ± 22.55	44.66 ± 29.93	49.82 ± 30.84	<0.001
CS	−15.43 ± 6.25	−19.05 ± 5.97	−19.86 ± 7.96	−21.52 ± 7.15	<0.001
LS	−20.68 ± 12.38	−20.89 ± 12.34	−20.27 ± 11.09	−30.30 ± 17.36	<0.001
**Apical**	**High T2 (+) (n = 27)**	**High T2 (−) (n = 39)**	**High T2 (+) (n = 13)**	**High T2 (−) (n = 149)**	**P value**
Thickness	14.96 ± 3.06	15.16 ± 3.30	11.01 ± 3.89	11.23 ± 3.83	<0.001
RS	33.37 ± 17.26	26.38 ± 13.39	49.12 ± 15.65	47.52 ± 27.07	<0.001
CS	−17.76 ± 5.39	−15.53 ± 5.10	−22.66 ± 4.92	−21.33 ± 6.88	<0.001
LS	−23.26 ± 14.53	−20.01 ± 12.20	−23.51 ± 11.00	−35.26 ± 21.08	<0.001

HighT2: high signal intensity on T2 weighted imaging; LGE: late gadolinium enhancement; RS: radial strain; CS: circumferential strain; LS: longitudinal strain.

**Figure 4:**
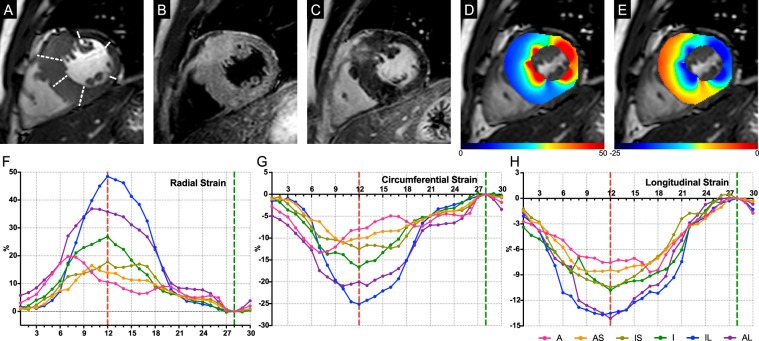
A 30-year-old male HCM patient. Hypertrophic segments of the mid-ventricular level, including the anterior (**A**), anterior septum (AS), inferior septum (IS), and inferior (I) wall, while the inferior lateral (IL) and anterior lateral (AL) wall thicknesses were less than 15 mm. Focal LGE and HighT2 coexistence were clearly displayed in the anterior wall. Strain impairment was present in the A, AS, IS and I segments. (**A**) End-diastolic phase of cine. (**B**) T2-STIR image. (**C**) PSIR image. (**D**) Radial strain at end-systolic phase. (**E**) Circumferential strain at end-systolic phase. (**F–H**) Radial, circumferential and longitudinal strain of the 6 segments at the mid-ventricular level.

Multivariate linear regression results are shown in Table 5. The segment thicknesses affected strain parameters in 3 directions. LGE presences are independent predictors of reduction in LS of all segments and segments <15 mm. HighT2s are independent predictors of reduction in CS and LS of segments <15 mm. Radial, circumferential and longitudinal segmental strain of one case are shown in Fig. 4.

**Table 5:** Results of multiple linear regression analysis.

	Parameter	All segments		Segments ≥15 mm (n = 231)		Segments ≤15 mm (n = 681)	
		beta	P value	beta	P value	beta	P value
RS	Thickness	−0.528	<0.001	−0.250	<0.001	−0.479	<0.001
	HighT2	−0.016	0.602	0.102	0.162	−0.067	0.063
	LGE	−0.060	0.067	0.055	0.444	−0.044	0.248
CS	Thickness	0.550	<0.001	0.230	<0.001	0.452	<0.001
	HighT2	0.047	0.120	−0.026	0.725	0.101	0.006
	LGE	0.017	0.607	−0.132	0.069	0.030	0.434
LS	Thickness	0.362	<0.001	0.144	0.031	0.357	<0.001
	HighT2	0.041	0.219	−0.046	0.537	0.091	0.016
	LGE	0.127	<0.001	0.079	0.285	0.113	0.005

## Discussion

Both cellular and extracellular pathological processes are observed in HCM. It is possible to evaluate replacement fibrosis with LGE, the gross macroscopic abnormalities would be reflected by segment thickness, while the cellular and extracellular compartments at the microscopic level could be assessed by ECV and native T1 values. In HCM patients, a strong correlation was reported between the ECV value and extent of LGE^20^, and a much weaker correlation between the native T1 and LGE was observed^21^. However, contrast agents are required to measure both LGE and ECV values.

Interestingly, Abdel-Aty *et al*.^22^ indicated that focal high signal intensity (SI) areas in T2-weighted CMR are common in patients with HCM and are mostly but not exclusively related to irreversible myocardial injury. Melacini *et al*.^23^ performed T2-weighted sequences for edema and late enhancement (LE) sequences. In their study, edema was present in 24/44 (54%) patients, while LE, edema and hypoperfusion presented in corresponding segments. It has been concluded that areas with HighT2 might be suggestive of myocardial oedema as a result of myocardial ischaemic injury, which is representative of a more active disease state in patients with HCM.

Recently, some researchers have investigated whether HCM patients with high T2 show increase in the arrhythmic burden^24–26^ and sudden cardiac death rate compared with patients without HighT2^18^. Cardiac troponin, a biomarker of myocardial injury, has been shown to be correlated not only with HighT2 extent^19^ but also with GLS in echocardiography studies^27^. In this context, it has been hypothesized that HighT2 might impact LV strain as well as LGE.

The effects of both LGE^28–30^ and elevated ECV^17^ impact LV deformation. In our HCM population, patients with weightier LV mass and LGE extent would have more serious impairment of RS, CS and LS. This phenomenon is consistent with previous studies. At the global level, GRS, GCS and GLS were all significantly correlated with HighT2 extent, but it was not sufficient to clarify that HighT2 impairs LV deformation. In addition, the HighT2 extent is correlated with LGE extent and LV mass itself^22^. Thus, segmental analysis was needed to prove this finding.

The strain parameters were significantly correlated with segment thicknesses. However, there is no significant thickness differences between the HighT2 presence with LGE presence and non-presence group. In the two groups with LGE, the HighT2 presence showed a significant difference in CS only at the mid-ventricular level. In the groups without LGE, the HighT2 presence showed a significant difference in LS only at the mid-ventricular level. The statistical differences were both concentrated in mid-ventricular level. HighT2 and LGE presences involved the mid-ventricular levels slightly more than basal and apical levels would be the possible reasons. In the multivariable regression analysis, segment thicknesses were the determinant predictors of strain impairment of all directions. LGE presences were independent predictors of reduction in LS of all segments and segments <15 mm. HighT2s were independent predictors of CS and LS reduction only in segments <15 mm. HighT2 presences, usually considered as edema, were recent pathological changes. The possible reason that the influence of edema to strain would be not as important as thickness especially in thickening segments.

T2-weighted imaging with fat saturation is known as an oedema-sensitive CMR sequence. In the Lake Louise Criteria, HighT2 was one of the diagnostic items of myocardial inflammation^31^. Myocarditis patients showed reduced RS, CS and LS values compared with those of healthy controls^32^. T2-STIR was proved to accurately detect acute myocardial infarction and identify the salvage area^33^.

In HCM, the HighT2 area is mostly localized with LGE in the mid-wall of the myocardium. The mechanism for regional T2-signal enhancement in HCM is still unknown. However, several theories could explain these phenomena. High T2 SI has been related to myocardial tissue oedema, which is due to myocarditis and acute ischaemia and may reflect an atypical distribution of one or both two-pathophysiology statuses. This finding would indicate a potential for high T2 SI to be a biomarker of different extents of disease activity in HCM. In the segmental analyses of our study, a large proportion of HighT2 segments were accompanied by LGE (126/178). This ratio is close to that found in a previous study^19^. LGE is detected by CMR in most HCM patients. Furthermore, the presence of LGE was considered to be fibrosis and added to the risk of sudden death. However, LGE could not distinguish recent myocardial infarction from chronic infarction in HCM. RS, CS and LS were all reduced in LGE segments compared with non-LGE segments. HighT2 with LGE in HCM might indicate active tissue injury with elevated cardiac troponin. The minority of segments (52 segments) presented with HighT2 alone. Finally, although the average segment thickness was close to that of negative segments, reduced LS was observed.

## Limitations

Several limitations of the current study should be mentioned. First, our conclusion needs confirmation within larger prospective multicentre studies, as this was a single-centre cohort setting. Second, although subtle influences of the High T2 to LV strain were observed, the pathology and nature of HighT2 in HCM is not well understood. Third, there are no follow-up data to observe the influence of HighT2 presence on LV deformation. Fourth, the valuation of HighT2 and LGE was completed by two observers, but subjectivity still cannot be avoided. Quantification mapping techniques such as T2 mapping and ECV calculating are still need to be used to verify the conclusion. Finally, no control individuals were involved in the current research because the aim of this study was to explore the relative distribution and prevalence of abnormal T2-weighted image (T2WI) findings among the confirmed HCM patients; we did not aim to identify the high T2WI prevalence relative to normal populations. Notably, high T2WI SI is reported to be rare in normal populations.

## Conclusions

In this study, the HighT2 extent in HCM was observed to be correlated with global strain parameters. At the segmental level, LGE segments with HighT2 appeared to have lower CS values, and non-LGE segments with HighT2 showed lower LS at the mid-ventricular level. In the multivariable regression analysis, HighT2 were independent predictors of reduction in CS and LS of segments <15 mm. HighT2 indeed affects LV deformation, and follow-up studies are still warranted.
